# Inclusive Contactless Monitoring for Older Adults From Diverse Backgrounds: Mixed Methods Study

**DOI:** 10.2196/79892

**Published:** 2026-07-03

**Authors:** Titilola Yakubu, Nooshin Jafari, Samya Torres, Michael Lim, David Rivest-Henault, Thomas Vaughan, Catherine Proulx, Linda Pecora, Di Jiang, Kendall Ho

**Affiliations:** 1 Faculty of Medicine, University of British Columbia, 6200 University Blvd, Vancouver, BC, V6T 1Z4, Canada, 1 604 822 0327; 2 Medical Devices Research Centre, National Research Council Canada, Boucherville, QC, Canada

**Keywords:** contactless monitoring, vital signs, digital intervention, remote monitoring, older adults, telemedicine

## Abstract

**Background:**

As Canada’s population ages, accessible tools for chronic health monitoring are increasingly needed. Traditional and contact-based devices pose barriers for underserved populations due to cost, maintenance, and usability. Contactless sensing technologies offer a promising alternative, but equitable development requires inclusive engagement and diverse data collection.

**Objective:**

This study aimed, first, to gather perspectives from culturally and health-diverse older adults on innovative contactless vital sign monitoring technology and, second, to build on a diverse dataset for developing and testing the contactless sensing software VitalSeer by the National Research Council of Canada.

**Methods:**

A mixed methods study was conducted with older adults from diverse backgrounds across 3 sites. Participants were asked to respond to a questionnaire that had closed- and open-ended questions regarding their perspectives on contactless sensing technology. Video and reference vital sign data were collected from each participant using a portable system designed specifically for gathering controlled data.

**Results:**

We collected data from 48 participants (mean age 70, SD 8 years), of whom 98% (n=47) expressed a positive perception of the usefulness of a contactless sensing system. We also identified four themes from the qualitative analysis of the open-ended questions: (1) perceived value—system potential and clinical relevance; (2) ease of use—noninvasiveness and comfort; (3) trust and transparency—data security and clarity of design; and (4) inclusion and improvement—accessibility, functionality, and feature expansion. Finally, the collected data, 288 minutes of concurrent video and reference vital sign data, will be used to test and enhance contactless sensing software for diverse older adult populations.

**Conclusions:**

This work advances the goal of inclusive medical device research and development. It highlights the potential for contactless sensing to be adopted to support independent living for older, diverse adults. Research is ongoing to adapt the technology for widespread adoption.

## Introduction

### Background

As Canada’s population ages, the demand for health care services, particularly those aimed at monitoring and managing chronic health conditions, is growing [1]. Traditional monitoring solutions require in-person checkups, which can be physically exhausting and disruptive [2,3]. Current contact-based solutions, such as using a pulse oximeter, might be undesirable or less suitable for some populations. Limiting factors include the cost of purchase, the need to manage an extra device, and the need to properly maintain and use it regularly. Older adults, particularly those living independently in underserved populations, may not have access to adequate tools for monitoring their health, putting them at greater risk [4].

Research has found that well-structured community engagement efforts, developed through meaningful consultation and active participation, can lead to positive changes in health behaviors, better access to health care services, enhanced health literacy, and improved health outcomes [5]. Integrating diverse perspectives in health technology development is crucial to address disparities in health outcomes, barriers to service access, and representation gaps [6]. Historically, marginalized and underserved populations have been excluded from health technology development, leading to poor uptake and even technological bias within important health assistive devices [7,8]. Additional barriers, such as cost, maintenance, and unclear added value, further deter widespread use among these demographics [9].

VitalSeer [10,11] is a software technology under development by the National Research Council of Canada for contactless, noninvasive vital signs assessment. It uses camera-based remote photoplethysmography (rPPG) to measure heart rate, respiratory rate, and oxygen saturation (SpO_2_) [12]. This technology could enable an easy-to-use monitoring solution that uses ubiquitous cameras, for example, mobile phone cameras, webcams, or home surveillance cameras. Sensing based on rPPG relies on the analysis of the light reflected by superficial tissues through the skin, so it can be affected by several extrinsic and intrinsic factors, such as the quality of the camera, skin type and tone, and environmental lighting conditions. Therefore, representative data acquired from a diverse population are critical to ensuring applicability.

The purpose of this study was to engage diverse populations for the development of contactless sensing technology [10] and to gather pertinent perceptions for technology improvement and eventual deployment. The contributions of this work include (1) a report on the perceptions of contactless sensing technologies from diverse populations in real-world community settings and (2) the acquisition of diverse high-quality data for VitalSeer development and testing.

### Objective

The primary objective of this study was to gather perceptions about technology adoption from older adults with diverse health and cultural backgrounds. In parallel, a secondary objective was to collect high-quality diverse data to support the development of the technology.

## Methods

### Study Design

We conducted a mixed methods study using a concurrent triangulation design, in which closed-ended questions and open-ended questions were administered to the same convenience sample of older adults living in British Columbia, Canada. Quantitative and qualitative data were collected concurrently during the same study sessions, analyzed independently, and then integrated during the interpretation phase. Quantitative findings were used to identify patterns and trends, while qualitative responses were used to contextualize and explain those themes, with convergence across both datasets used to strengthen our conclusions.

### Ethical Considerations

Ethical approval for the study was obtained from the University of British Columbia Ethics Board (H23-02571) and the National Research Council of Canada (NRC 2020‐134).

All participants provided informed consent before participating in the study. Initial consent discussions were conducted via telephone calls and in person to explain the study objectives and address participants’ questions.

All study data were deidentified before analysis by replacing identifiable information, such as names, with participant IDs. Study data were stored securely, and only study team members could access them.

Participants received a CAD $10 (CAD $1=US $0.71 as of June 17, 2026) e-gift card as a token of appreciation for their time and participation.

### Sampling and Recruitment

Participant recruitment was facilitated through REACH BC, an online research platform for the Canadian province of British Columbia. Additionally, the research team conducted extensive outreach to multicultural older adult home and community centers in the Lower Mainland region of Vancouver, British Columbia. Out of 49 centers approached, 2 (4.1%) were selected for facilitating on-site participant recruitment and study conduct: Unique Get Together Society and South Granville Seniors Centre. The centers were chosen based on their representation of a diverse range of ethnic groups, including Black, Latino, White, and Chinese communities. Furthermore, interest and capacity to participate, proximity to the research lab, and the availability of suitable space were also factors considered for selection.

Eligibility criteria prioritized older adults aged 60 years and older, who could attend a 1-hour in-person session at participating community centers. Participants younger than 60 years were allowed to participate for broader inclusion across cultural and ethnic backgrounds (people of color and Indigenous communities). Participants were required to remain still for approximately 2 minutes at a time. Proficiency in English was required unless a multilingual member of the research team was available to assist with instructions. Furthermore, participants needed to be in generally good health and capable of sitting still. The study emphasized the inclusion of individuals from diverse backgrounds, with varying skin tones and health conditions, to ensure a representative sample. Individual sessions were conducted at either a laboratory or one of 2 community centers.

### Data Collection

#### Recordings

**Figure 1.**
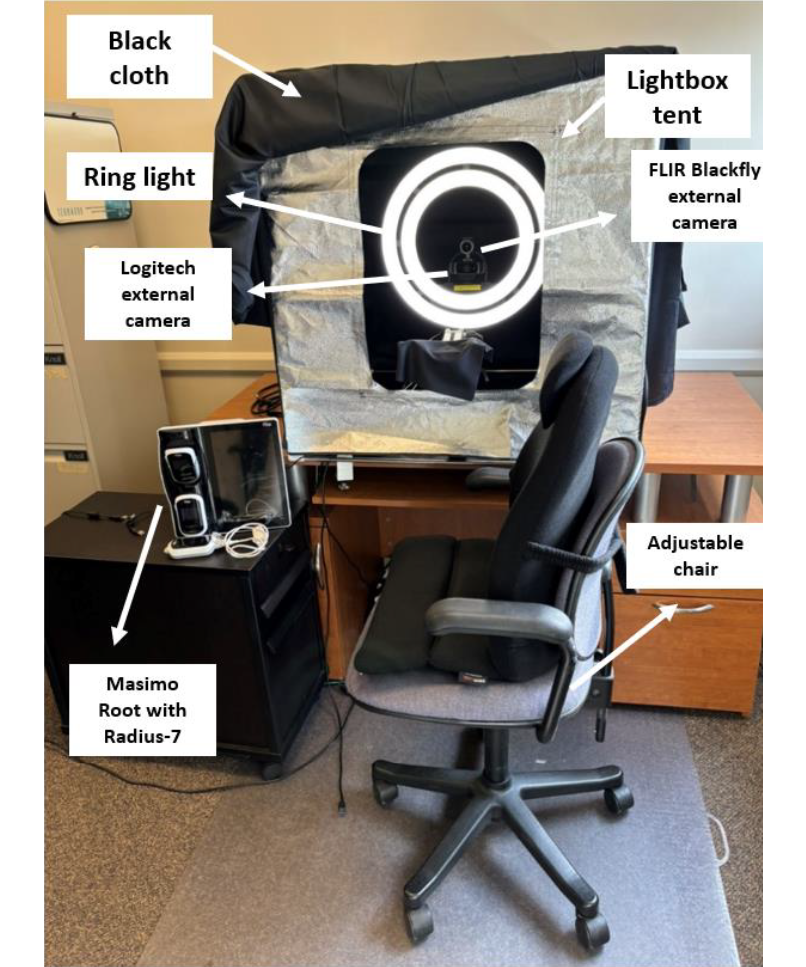
Hardware configuration of the data collection system at the research team’s laboratory.

A deployable system, henceforth referred to as a “lightbox,” was designed specifically for controlled data collection in this study. The lightbox featured an LED ring-light enclosure for uniform illumination, a chin rest to help participants remain still and minimize motion during video acquisitions, a metal support structure to mount 2 cameras (a consumer-grade Logitech webcam and a research-grade FLIR Blackfly), both directed at the participant’s face, and a black cloth to cover the participant and block external light from entering. The lightbox was specifically designed to help collect data under optimal and reproducible conditions, ensuring minimal influence by external variables, such as fluctuations in ambient light and participant movement. Adjacent to the lightbox was a Lenovo T470s laptop (Windows 10) running the VitalSeer software. The cameras were connected via USB, and the laptop was connected to Ethernet for optimal bandwidth, as depicted in Figure 1.

Reference physiological parameters were measured from Food and Drug Administration–cleared and Health Canada–approved Masimo medical contact-based patient monitoring devices. These devices included a reference pulse oximeter, which provides noninvasive measurements of heart rate, respiratory rate, and SpO_2_, and the Masimo Root monitor equipped with the Radius-7 continuous monitoring system (Masimo Corp). The Masimo Root system uses advanced signal extraction technology to deliver highly accurate vital sign measurements even in challenging clinical environments, such as low perfusion or motion artifacts.

Participants underwent three 2-minute recordings. Simultaneous readings from the Masimo Root system served as the gold standard for comparison, as these data were later used for validation.

#### Questionnaire

The questionnaire assessed user perceptions, acceptance, and usability of the remote health monitoring system using Likert-scale and open-ended questions. It evaluated perceived usefulness for self-monitoring and remote health care, willingness to share data, comfort with prolonged use, and concerns about wearables. Open-ended questions gathered feedback on user experience, system improvements, and overall satisfaction. This approach provided insights into user needs and barriers, informing system refinements for real-world application.

The questionnaire was administered at the end of the session after the VitalSeer recordings were completed. The survey contained a mix of quantitative and qualitative elements, including 6 rating scale questions and 4 open-ended questions to allow participants to provide more detailed feedback (Table 1). Skin tone scale data were also collected during study sessions using the Fitzpatrick skin type classification system [13]. Participants self-identified their skin type using the Fitzpatrick scale (I-VI), and a research team member (TY, NJ, or ST) also independently categorized each participant based on visible facial skin characteristics. In cases of discrepancy, the team revisited the classification and assigned the closest matching Fitzpatrick skin type.

**Table 1.** Participants’ postsession questionnaire.

Question text	Type
I feel that the system that collects my vital signs would be useful to me in self-monitoring my health by frequently tracking of my vital signs at home	Likert scale (5-point)
I feel that the system that collects vital signs would be helpful for healthcare providers to monitor their patients remotely (*e.g*., for patients living in rural/remote areas)	Likert scale (5-point)
In the future, I would be willing to send my health-related data and share my results from the system to my healthcare provider online	Likert scale (5-point)
In the future, I would be willing to use the system for a longer duration (*e.g.,* one week) to track my health-related data, after my visit to emergency department or hospital	Likert scale (5-point)
In the future, I think the system will be helpful to me for managing the health-related concerns related to wearable sensors that touch skin and are used between multiple users	Likert scale (5-point)
In the future, I think the system at home will be helpful for me to self-manage my health and live more independently	Likert scale (5-point)
After testing the system, what do you think about it? (What did/did not like about it)?	Open-ended
Do you have any suggestions for improving the system?	Open-ended
Do you have any suggestions for us to help improve your experience?	Open-ended
On a scale of 1-10, how was your overall test experience with the system today? Would you like to elaborate?	Open-ended

### Data Processing and Analysis

Quantitative data were analyzed descriptively using SPSS Statistics for Mac (Version 29.0; IBM Corp). Frequencies and percentages were calculated for Likert-scale responses, and demographic variables were summarized using descriptive statistics (frequencies and proportions).

Qualitative feedback from the open-ended questions was analyzed using NVivo 12 (Lumivero) following reflexive thematic analysis. The analysis involved familiarization with the data through repeated reading of responses, inductive code generation, theme development, theme review and refinement, and final theme definition and naming. Codes were generated inductively from participant responses rather than a predefined coding framework, allowing themes to emerge directly from the data. Two coders (TY and NJ) independently reviewed and coded the responses, comparing findings throughout to enhance consistency and credibility. In cases of disagreement, coders collaboratively reviewed the relevant data and reassessed their interpretations. Where consensus could not be reached, KH reviewed the codes within the context of the research questions and made the final decision.

### Ensuring Rigor and Trustworthiness

We ensured the rigor and trustworthiness of our research through triangulation, combining closed-ended responses and open-ended responses to capture and explore deeper perspectives. Peer debriefing by 2 independent coders (TY and NJ) validated themes, ensuring consistency and accuracy.

We also documented each research step to maintain transparency and reduce bias, enhancing credibility. The team’s positionality also strengthened the process: KH, with more than 25 years of experience in mixed methodologies, provided theoretical expertise; TY, who is experienced in qualitative methods and has an MBBS, contributed clinical insights and methodological knowledge; and NJ, a researcher with an engineering background, added relevant academic and practical experience.

## Results

### Participants

**Figure 2.**
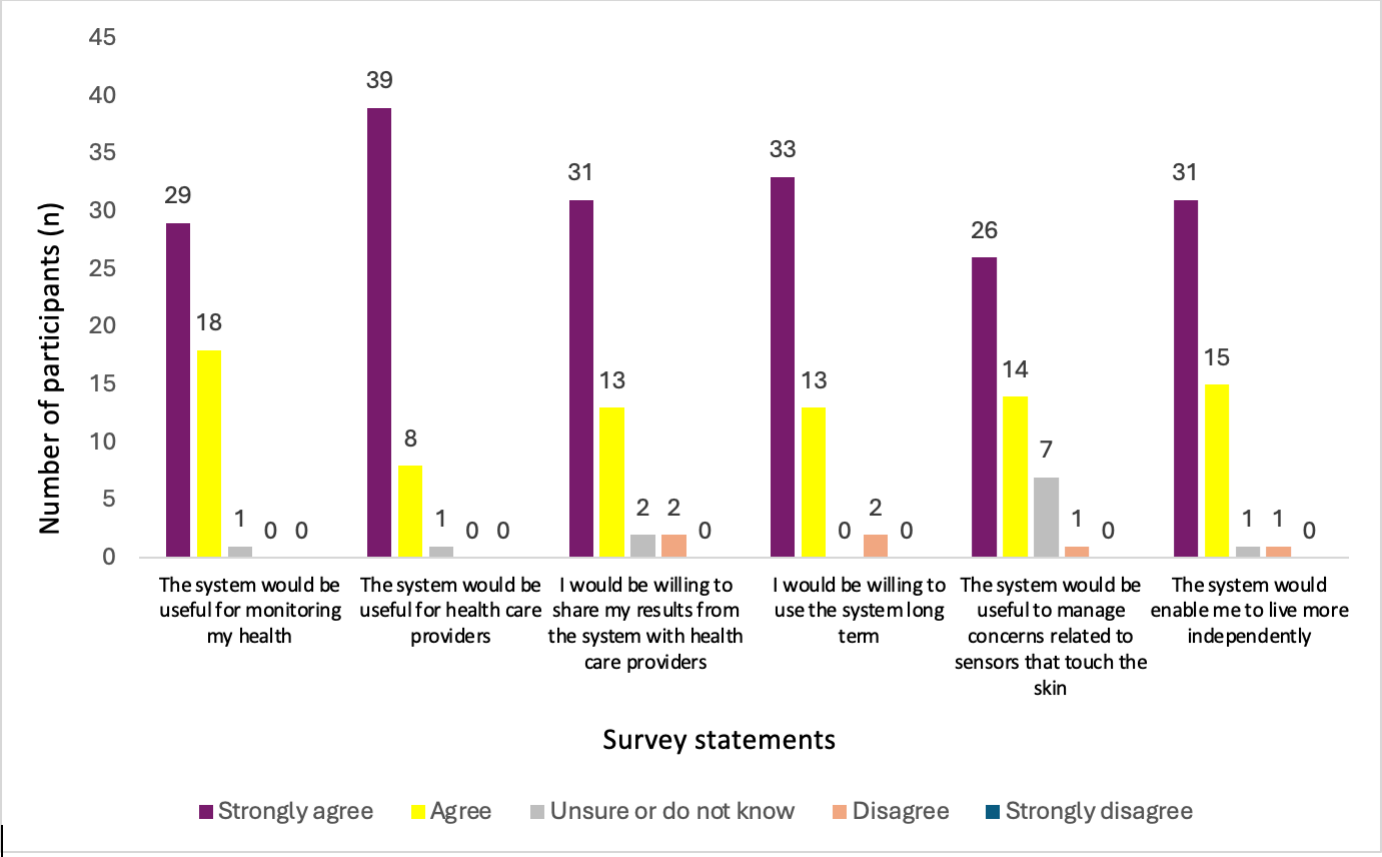
Responses to Likert-scale questionnaire items regarding perceptions of the contactless monitoring system.

We conducted a focused community recruitment phase over 34 days (March 2-April 4, 2024), following several months of engagement with community stakeholders. A total of 48 participants were recruited, with 26 sessions held at community centers. Most participants were aged between 66 and 70 years. The mean age of participants was 70 (SD 8) years. Detailed demographic information, including age, sex, data collection location, and Fitzpatrick skin tone [13] (as observed by the study team), is presented in Table 2 and Figure 2. The study also included 5 participants aged 60 or younger, comprising 4 Black individuals and 1 Indigenous participant.

**Table 2.** Participant demographics (N=48).

Demographics	Participants, n (%)
Age group (y)	
<55	1 (2.1)
56‐60	4 (8.3)
61‐65	8 (16.7)
66‐70	14 (29.2)
71‐75	5 (10.4)
76‐80	8 (16.7)
≥81	8 (16.7)
Sex	
Female	29 (60.4)
Male	19 (39.6)
Location	
UBC^a^ Digital Emergency Medicine Laboratory	22 (45.8)
Unique Get Together Society	13 (27.1)
South Granville Senior Center	13 (27.1)
Fitzpatrick skin tone scale, type I	3 (6.25)

a UBC: The University of British Columbia.

### Recordings

Each of the 48 participants contributed 3 rounds of recordings, resulting in 144 distinct recordings. Each of these recordings consisted of paired video data (from 2 cameras) over 2 minutes, yielding 288 minutes of high-quality, controlled video data for each of the 2 cameras. In addition, reference vital sign data were collected from Food and Drug Administration–cleared and Health Canada–approved Masimo medical contact-based patient monitoring devices that correspond to the videos.

### Questionnaire

#### Likert-Scale Questions

Figure 2 shows that most (47/48, 98%) participants agreed that contactless monitoring would be useful for independently monitoring their vital signs. Participants largely agreed on several other points, including the willingness to share results with health care providers, long-term use, and benefits for independent living.

However, 8% (4/48) of participants disagreed or were unsure about sharing their results with health care providers, 4% (2/48) disagreed with using the system long term, and 2% (1/48) felt that the system would not help manage concerns related to contact monitoring or support independent living.

#### Open-Ended Questions

##### Overview

We asked participants open-ended questions to elicit further perceptions of the technology. We identified four overarching themes that reflect participants’ perceptions of the system: (1) perceived value—system potential and clinical relevance; (2) ease of use—noninvasiveness and comfort; (3) trust and transparency—data security and clarity of design; and (4) inclusion and improvement—accessibility, functionality, and feature expansion.

Each theme is broken down into subthemes, with participant quotes used to illustrate key points.

##### Theme 1: Perceived Value—System Potential and Clinical Relevance

###### Value for Remote Monitoring in Chronic Conditions

Participants emphasized the potential of VitalSeer to support at-home health monitoring, especially for individuals with chronic or respiratory conditions. Many saw it as a valuable tool that could allow early detection of symptoms and continuous monitoring without the need for frequent clinic visits. They believed this would be particularly useful for patients with long-term conditions who often require close surveillance:

This system has a lot of potential, especially for people with breathing problems or conditions like COPD. It could help doctors catch issues early.

[Participant 12]

This could be a very useful tool for patients to use and for doctors to gain data on their patients. It is very promising even in this test configuration.

[Participant 31]

###### Anticipated Benefits for Patients and Clinicians

In addition to monitoring individual health, participants highlighted the broader benefit of the system in supporting clinical decision-making. They felt that providing real-time access to vital signs could enhance health care providers’ ability to respond quickly to changes in a patient’s health, enabling more efficient and personalized care. This was viewed as a win-win for both patients and clinicians:

If this works as intended, it’ll reduce my visits to the clinic and still help my doctor stay updated on my health.

[Participant 3]

The idea that doctors can see what’s going on in real-time from home—that’s huge.

[Participant 19]

###### Brief Positive Feedback Without Detailed Commentary

While the majority offered detailed responses, a small proportion (12.5%) simply described the system as “fine,” “good,” or “satisfactory” without elaboration. This may reflect satisfaction without specific points of critique, or it may indicate a neutral experience with no strong positive or negative reactions. Although not descriptive, this feedback suggests the system was at least acceptable in its current form:

Good.

[Participant 38]

Fine. It worked okay.

[Participant 40]

### Theme 2: Ease of Use—Noninvasiveness and Comfort

#### Simple and Fast to Use

A key feature appreciated by participants was the system’s simplicity. Many emphasized how quick and effortless it felt to use, with a minimal learning curve. This ease of use was seen as crucial for ensuring long-term engagement, especially for users with limited time or low interest in technology. The process was described as unobtrusive and easy to fit into everyday life:

It was easy and fast; I didn’t feel like it took much time out of my day.

[Participant 5]

Convenient and easy to navigate.

[Participant 13]

#### Low Technical Barriers

Even participants with limited prior experience using digital health tools commented on how straightforward the system was. They appreciated not having to deal with complicated instructions or setup steps. This accessibility was framed as especially important for broad adoption, including among older adults or individuals less familiar with technology:

Not complicated.

[Participant 37]

Effortless.

[Participant 29]

#### Noninvasive Experience

Participants highlighted that the system’s noncontact approach was one of its most appealing features. Unlike wearables or devices that require physical contact or attachment, for many, this hands-off approach appeared to be convenient for daily use:

The fact that it’s non-invasive makes it less stressful to use daily.

[Participant 43]

I like that it doesn’t require me to wear anything or have anything attached to my body.

[Participant 30]

### Theme 3: Trust and Transparency—Data Security and Clarity of Design

#### Concerns About Data Privacy and Protection

Several participants raised concerns about the safety of personal health data collected by the system. Given the sensitivity of the information involved, they wanted clear assurances that their data would be securely transmitted and stored. Participants highlighted this as a critical factor in determining their willingness to use the system regularly:

The big concern would be data security during transmission.

[Participant 26]

If I’m going to be using this regularly, I need to know that my data is safe.

[Participant 11]

#### Lack of Transparency About Data Use

Participants also expressed a desire for more transparency about how the data collected would be used. Specifically, they wanted to understand what types of data were being recorded, who would have access to them, and how they would inform their care. This was seen as important for building trust and encouraging users to engage with the system fully:

I want to know exactly what data is being collected and how it will help in the long run.

[Participant 30]

More explanation would help. It’s not very clear right now how this ties into my overall care.

[Participant 41]

#### Perception of an Unfinished Design

Some participants described the system as still in development and wanted clearer communication about its future direction. These participants noted a lack of polish in the current interface and requested updates on what improvements could be expected. They felt that greater transparency in the development process would improve their confidence in the system:

The design feels a bit unfinished. If we could get more information about where it’s heading, it would help us trust it more.

[Participant 21]

It looks like it’s still in progress. I'd like to see more polish before using it daily.

[Participant 8]

### Theme 4: Inclusion and Improvement—Accessibility, Functionality, and Feature Expansion

#### Barriers for Older Adults and Low-Tech Users

Participants raised concerns about the system’s accessibility for older adults or users unfamiliar with technology. They emphasized that many older adults might lack smartphones, tablets, or reliable internet access. These barriers could prevent widespread adoption unless targeted solutions, such as simplified interfaces or alternative access methods, were implemented:

This is not going to be feasible for adults who do not have access to a computer or cellphone. How are you going to solve that problem?

[Participant 9]

It’s a great idea, but I’m worried about how people with little tech knowledge will manage.

[Participant 24]

#### Need for Training and Visual Support

To support wider usability, participants recommended features such as color-coded feedback and audio instructions. These additions were viewed as particularly important for older adults, many of whom may struggle to interpret numerical health data. Suggestions included multilingual audio guidance and visuals that clearly indicate whether a measurement is within a healthy range:

Color coding for vital signs (numbers) so seniors will understand if the numbers are good.

[Participant 17]

Voice guidance in the app use—especially in multiple languages—would really help.

[Participant 23]

#### Desire for Expanded Monitoring Features

Several participants suggested that the system include other vital signs, such as blood pressure, SpO_2_, and temperature. These metrics were viewed as essential for people managing chronic illnesses—particularly in communities where conditions like hypertension are common:

Blood pressure in the Black community is important… hypertension is a typical problem, so consider that one in VitalSeer.

[Participant 38]

What would be useful would be a warning for low O_2_ saturation.

[Participant 46]

#### Suggestions for Practical Design Considerations

Participants also made comments about day-to-day use cases—such as whether the system would function properly if someone was wearing glasses or makeup. Others noted that low internet connectivity in rural or remote areas might prevent some users from accessing the system altogether:

At this time, it is OK to remove the glasses, but later, you need to consider not removing them.

[Participant 2]

This needs to work for rural users with slow or no internet.

[Participant 36]

## Discussion

### Principal Findings

This study demonstrates the feasibility of contactless sensing research and development in real-world community settings beyond the laboratory, engaging older adults from diverse backgrounds. The study participants included an older adult population comprising Black, Latino, White, Indigenous, and Chinese communities. Contactless sensing technology was well received, making it a promising solution for monitoring vital signs among older adults. Feedback from the participants provided insights into technology adoption.

### Comparison With Previous Work

Our findings also show interest in technological solutions to support independent living for older adults. Noninvasive features helped increase acceptance by overcoming a major barrier to technology adoption in older adults compared with previous studies [14-17]. Although several studies have explored the use of contactless health monitoring systems [18], few have advanced to real-world testing. Most research on rPPG has largely been limited to laboratory settings [19-21]. Our study implemented a field-based approach and revealed important insights into the practical challenges and adoption of the technology when used by diverse older adults. While participants raised comments related to ease of use and data privacy, security, and monitoring, these findings were consistent with findings from previous studies [16,22]. It will be important to identify, implement, and communicate measures that can help build trust in these technologies.

The data collection in this research also contributes to the development and robustness of the VitalSeer software through the inclusion of participants with varying skin tones, a known variable that affects the accuracy of rPPG-based systems [18,23]. The inclusion of diverse participants also reflects a commitment to equity, diversity, and inclusion in health care technology development. By ensuring representation across different skin tones, genders, and health conditions, this work promotes solutions that are accessible, effective, and beneficial to all.

### Novelty of This Clinical Study

One of the most important contributions of this study is its focus on diverse and underserved populations, addressing a significant issue in both health care and research: the underrepresentation of marginalized groups. By including a wide range of participants from various backgrounds, this study supports equitable health care solutions. Remote monitoring technologies have the potential to bridge gaps in health care access by providing individuals in underserved communities with tools to manage their health autonomously, particularly those who face barriers to frequent in-person medical visits. To our knowledge, this study is the first to elicit open-ended feedback from diverse participants to understand the perceived benefits and limitations of contactless sensing technology.

The use of contactless sensing technology in this study also shows that it can be implemented outside laboratory and traditional clinical environments, suggesting it can be made accessible to individuals who may not regularly interact with the health care system.

### Limitations

Camera-based vital sign monitoring is susceptible to poor lighting conditions [23,24]. In this study, we used the lightbox to control the environment and ensure high-quality data collection. The effect of lighting on rPPG quality is an area of continued research; the methods used for signal acquisition and analysis are expected to evolve and improve with time.

We cannot discount the possibility that the presence and use of the lightbox may have altered some participants’ perceptions of contactless sensing technology. If future researchers evaluate perceptions of a technological concept, it may be advisable to administer questionnaires before exposing participants to development or testing measures (in this study, the presence of the lightbox) that could influence their responses.

### Implications for Practice

The findings from this study have several implications for both health care practice and policy. First, the deployment of the contactless sensing technology in this study in community settings suggests that it could eventually be scaled up for broader use in home health monitoring programs, particularly for older populations. Such systems have the potential to ease the strain on health care facilities by empowering older adults to independently monitor and manage their health from home. This reduces the need for frequent hospital visits or long-term care stays, which is particularly beneficial in Canada, where rising emergency department wait times have been documented [18,23].

From a policy perspective, the results suggest that with the right design and implementation strategies, contactless monitoring systems can help close some of the gaps in health care access that disproportionately affect underserved populations. Future policy initiatives should focus on integrating these technologies into public health strategies, particularly in remote areas.

### Future Directions

Moving forward, several avenues for future research emerge from this study. First, insights and video data from this study will assist in the ongoing development of the VitalSeer software, including its current extension to the mobile environment. Future research could also focus on expanding participant pools to target, for example, individuals with severe health conditions. Participants with mobility impairments might provide valuable insights into how these technologies can be more effectively integrated. Finally, studies should explore the long-term impact of using contactless sensing technologies in home settings, particularly how their use influences health outcomes, quality of life, and health care use over time.

### Conclusions

In conclusion, contactless sensing represents a promising advancement in health monitoring. The development and testing we conducted in real-world settings, with a diverse sample population, highlight its potential to be successfully adopted to improve health outcomes and support aging in place for older adults. Further research and development are ongoing to ensure applicability under varying lighting conditions and the inclusion of additional features. These refinements will help facilitate its widespread adoption in Canada for both home and hospital use.

## Supplementary material

10.2196/79892 Multimedia Appendix 1 Questionnaire.

10.2196/79892 Checklist 1 SRQR checklist.

## Abbreviations

rPPG: remote photoplethysmography
SpO_2_: oxygen saturation
