# Supplementary material for: Inclusive Contactless Monitoring for Older Adults From Diverse Backgrounds: Mixed Methods Study
Source: JMIR Mhealth Uhealth. 2026 Jul 3;14:e79892. doi: 10.2196/79892 (PMC13330646; doi:10.2196/79892)
Supplement: Multimedia Appendix 1 [file mhealth-v14-e79892-s001.docx]

# Appendix 1. Questionnaire

**CASE REPORT FORMS:** CLINICAL DETERMINATION AND USABILITY TESTING FOR CONTACTLESS MONITORING AND REMOTE CARE FOR OLDER ADULTS

**Phase 2: Product Validation I**

**1. CASE REPORT FORM: Eligibility Screening and Study Status**

**1a. Participant contact details***:*

1. Participant name (first and last)
2. Participant preferred email contact
3. Participant preferred phone number contact
4. Participant preferred method of contact:
   1. Email
   2. Phone
   3. Other
5. Date of first contact
6. Referral source:
   1. REACH BC
   2. Word of mouth
   3. Other
7. Others: [open text for general notes]
8. Current status:
   1. Screening (eligibility pending)
   2. Eligible confirmed
      1. Enrolled
      2. Complete
   3. Ineligible confirmed
   4. Withdrew/ lost to follow-up

**1b. Inclusion and exclusion screening**

***Eligibility status***:

- Eligible (proceed with enrollment)
- Ineligible (stop further enrollment)
- Pending/ to be confirmed (follow-up to confirm eligibility)

***Inclusion criteria*** –prospective senior participants must meet all inclusion criteria to be eligible

- 60 years old + (per AiP guidelines)
- Able to attend a 1 hour in-person test session

***Exclusion criteria*** – the following would make prospective participants ineligible to participants (must answer yes to all items to participate)

- 1. able to read and understand English, unless suitable caregiver support is available to assist with study consent and procedures/ directions
  2. In general acceptable health
  3. able to sit still for approximately 2 minutes at a time

**1c. Form: Consent and enrollment details**

1. Participant consent obtained:
   1. Yes
   2. No
   3. Pending
2. Date of consent
3. Test session date:
   1. Confirmed
   2. Pending/ to be confirmed

**2. CASE REPORT FORM: PARTICIPANT INFORMATION**

1. Sex:
   1. Male
   2. Female
   3. Other
   4. Prefer not to answer
2. What language do you speak, read and write?

*Options*: Arabic, Cantonese, English, Filipino/Tagalog, French, German, Hindi, Indigenous language (please specify), Japanese, Korean, Mandarin, Other, Persian/Farsi, Prefer Not to Answer, Punjabi, Russian, Spanish, Urdu, Vietnamese

1. Do you identify with the following?

*Options*: Arab, Black, Chinese, Filipino, First Nations, Indigenous peoples of Canada, Inuit, Japanese, Korean, Métis, South Asian (e.g., East Indian, Pakistani, Sri Lankan, etc.), Southeast Asian (e.g., Cambodian, Laotian, Thai, Vietnamese, etc.), West Asian (e.g., Afghan, Iranian, etc.), White, Latin, Central, or South American (e.g., Brazilian, Chilean, Colombian, Mexican, etc.)

1. What city/town are you currently living in?
2. How would you describe your living arrangements?

*Options*: alone at home, with a partner (spouse), with caregiver, long-term care housing, private senior residence, etc.

1. How close are you to the nearest hospital/clinic?

*Example*: < 5km / 5-10km / 11-20km / 21km+

1. Are you wearing makeup?
   1. Yes
   2. No
   3. Prefer not to answer
2. Are you wearing:
   1. nail polish (expand on what color)
   2. makeup (expand)
   3. skincare product (expand)
3. Age (in year/month):
4. Height (in inches):
5. Weight (in pounds):
6. Smoking status:
   1. Current smoker, how many cigarettes/day?
   2. Former smoker
   3. Never smoked
   4. Prefer not to answer
7. How would you describe the skin on your face?

a. _____ Oily

b. _____ Normal

c. _____ Dry

d. _____ Not sure

e. _____ Prefer not to answer

f. _____Other (please specify)

1. Skin tone of participant based on the Fitzpatrick Scale:

a: Participant’s perception: [multiple choices]

- 1. Light, pale white
  2. White, fair
  3. Medium, white to olive
  4. Olive, moderate brown
  5. Brown, dark brown
  6. Black, very dark brown to black


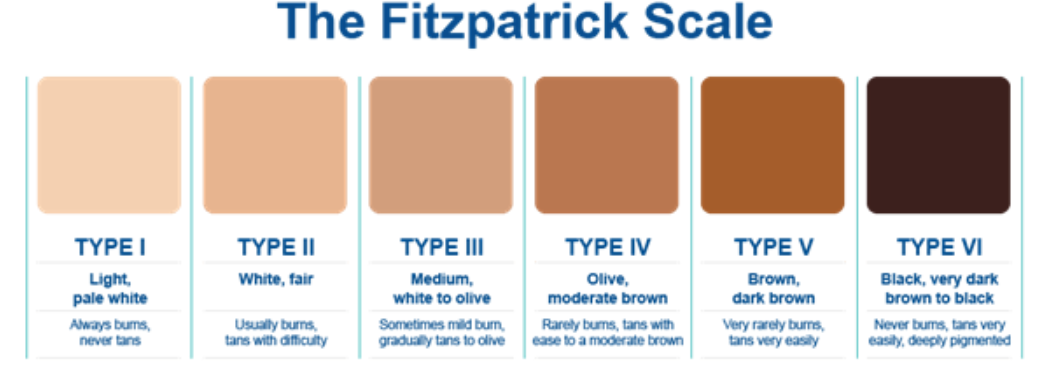


b: Researcher’s perception: [multiple choices]

- 1. Light, pale white
  2. White, fair
  3. Medium, white to olive
  4. Olive, moderate brown
  5. Brown, dark brown
  6. Black, very dark brown to black

1. Any medical condition or medical treatment you are currently undergoing? (add check boxes?)
   - Anemia.
   - Asthma.
   - Bronchitis.
   - Chronic obstructive pulmonary disease (COPD).
   - Congenital heart defects.
   - Congestive heart failure.
   - Emphysema.
   - Pneumonia (bacterial and viral)
   - Other: _______________________________
2. Any other relevant details provided by participant that may impact physiological parametric readings (ex. facial markings; other conditions):

**3. CASE REPORT FORM: SENSOR DATA COLLECTION**

**3a. Test session details**

*Repeated measure:*

1. Test location
2. NRC product version number/ release
3. Logitech Camera Model Version
4. FLIR Camera Model Version
5. Reference/gold standard equipment make/model

**3b. Product testing results**

Patient Identifier: ___________________

| Subject name or ID # | |  | | Date |  |
| --- | --- | --- | --- | --- | --- |
| *Time on mobile tablet/phone (<https://time.is/> ) : | | | | Exact or time difference if > 0.5sec | |
| *Time on computer / laptop (<https://time.is/>) : | | | | Exact or time difference if > 0.5sec | |
| Ring light intensity level | |  | Lighting type |  | |
| Run # | Time Started | Notes | | | |
|  |  |  | | | |
|  |  |  | | | |
|  |  |  | | | |
|  |  |  | | | |

1. **Form: Usability feedback**

|  | Strongly Agree  (5) | Agree  (4) | Unsure/ don’t know  (3) | Disagree  (2) | Strongly Disagree  (1) |
| --- | --- | --- | --- | --- | --- |
| 1. I feel that the set-up that collects my vital signs would be useful to me in self-monitoring my health by frequently tracking of my vital signs at home. |  |  |  |  |  |
| 1. I feel that the set-up that collects vital signs would be helpful for healthcare providers to monitor their patients remotely (e.g. for patients living in rural/remote areas). |  |  |  |  |  |
| 1. In the future, I would be willing to send my health-related data and share my results from the set-up to my healthcare provider online. |  |  |  |  |  |
| 1. In the future, I would be willing to use the set-up for a longer duration (e.g. one week) to track my health-related data, after my visit to emergency department or hospital. |  |  |  |  |  |
| 1. In the future, think the set-up will be helpful to me for managing the health-related concerns related to wearable sensors that touch skin and are used between multiple users |  |  |  |  |  |
| 1. In the future, think the set-up at home will be helpful for me to self-manage my health and live more independently |  |  |  |  |  |

| **Observations and general comments** |  |
| --- | --- |
| After testing our setup, what do you think about it?(What did/did not like about it)? | |
| Do you have any suggestions for improving the set-up? | |
| Do you have any suggestions for us to help improve your experience? | |
| On a scale of 1-10, how was your overall test experience with the set-up today? Would you like to elaborate? | |

**Thank you for your time!**
