# Supplementary material for: Inclusive Contactless Monitoring for Older Adults From Diverse Backgrounds: Mixed Methods Study
Source: JMIR Mhealth Uhealth. 2026 Jul 3;14:e79892. doi: 10.2196/79892 (PMC13330646; doi:10.2196/79892)
Supplement: Checklist 1 [file mhealth-v14-e79892-s002.pdf]

SRQR – Standards for Reporting Qualitative Research Checklist

Item	Description	How This Study Addresses It
**1. Title**	Identify study as qualitative or containing qualitative components	Title indicates "Perspectives," and Methods specify mixed-methods including qualitative analysis.
**2. Abstract**	Summary of background, purpose, methods, results, conclusions	Abstract now includes aims, qualitative thematic results, methods, and implications.
**3. Problem formulation**	Description of problem, significance, and review of relevant theory/research	Introduction discusses inequities in digital health, contactless sensing gaps, and need for diverse representation.
**4. Purpose / Research question**	Clear qualitative aims/questions	Aim: gather perceptions of older adults on contactless sensing.
**5. Qualitative approach & research paradigm**	Specify approach (e.g., thematic analysis), paradigm (e.g., constructivist)	Inductive thematic analysis; constructivist orientation implied (focus on participant perspectives).
**6. Researcher characteristics & reflexivity**	Roles, backgrounds, assumptions, and influence	Coders' backgrounds described; reflexivity acknowledged during coding and consensus discussions.
**7. Context**	Setting/site and contextual factors	Data collected in two multicultural community centers and a university lab.
**8. Sampling strategy**	How participants were chosen + rationale	Convenience + purposive sampling to ensure cultural, skin tone, and health diversity.
**9. Ethical issues**	Approvals and consent	UBC and NRC ethics approvals; written consent obtained.
**10. Data collection methods**	How data were collected, including changes during study	Open-ended questions administered after technology testing using a structured questionnaire.
**11. Data collection instruments & technologies**	Survey guides, questions, software used	Investigator-developed questionnaire with open-ended items; full instrument provided in Appendix A.
**12. Units of study**	Sample size, characteristics	48 adults (mean age 70), diverse ethnicities, Fitzpatrick tones I–VI.
**13. Data processing**	Transcription, data entry, management, security	Responses recorded directly in written format; managed and coded in NVivo 12; data deidentified.
**14. Data analysis**	How themes were developed, who was involved	Two coders independently performed inductive thematic analysis; consensus achieved through iterative meetings.
**15. Techniques to enhance trustworthiness**	Credibility, dependability, confirmability	Investigator triangulation, peer debriefing, audit trail, reflexive discussions; aligned with SRQR.
**16. Findings / Results**	Themes with supporting data	Four main themes with representative participant quotes.

Item	Description	How This Study Addresses It
**17. Synthesis & interpretation**	Meaning of findings, relation to literature	Discussed in context of prior digital health adoption and rPPG research.
**18. Links to empirical data**	Direct quotes	Multiple participant quotes included for every theme/subtheme.
**19. Integration with prior work**	How findings compare to previous research	Comparison highlights novelty (community-based diverse sample) and aligns with known issues around usability and trust.
**20. Limitations**	Methodological or analytic limitations	Discussed (lighting, lightbox influence, not aiming for saturation, convenience sampling).
**21. Implications & transferability**	Practical significance + areas for future research	Implications for equitable technology design, trust-building, and mobility-friendly deployment discussed.
